# Epidemiology of Out-of-Hospital Cardiac Arrest Due to Suffocation Focusing on Suffocation Due to Japanese Rice Cake: A Population-Based Observational Study From the Utstein Osaka Project

**DOI:** 10.2188/jea.JE20160179

**Published:** 2018-02-05

**Authors:** Kosuke Kiyohara, Tomohiko Sakai, Chika Nishiyama, Tatsuya Nishiuchi, Yasuyuki Hayashi, Taku Iwami, Tetsuhisa Kitamura

**Affiliations:** 1 Department of Public Health, Tokyo Women’s Medical University, Tokyo, Japan; 2 Department of Traumatology and Acute Critical Medicine, Osaka University Graduate School of Medicine, Osaka, Japan; 3 Department of Critical Care Nursing, Kyoto University Graduate School of Human Health Science, Kyoto, Japan; 4 Department of Acute Medicine, Kindai University Faculty of Medicine, Osaka, Japan; 5 Senri Critical Care Medical Center, Osaka Saiseikai Senri Hospital, Osaka, Japan; 6 Kyoto University Health Service, Kyoto, Japan; 7 Division of Environmental Medicine and Population Sciences, Department of Social and Environmental Medicine, Graduate School of Medicine, Osaka University, Osaka, Japan

**Keywords:** out-of-hospital cardiac arrest, suffocation, Japanese rice cake, outcome

## Abstract

**Background:**

Japanese rice cake (“*mochi*”) is a major cause of food-choking accidents in Japan. However, the epidemiology of out-of-hospital cardiac arrests (OHCAs) due to suffocation caused by rice cakes is poorly understood.

**Methods:**

OHCA data from 2005 to 2012 were obtained from the population-based OHCA registry in Osaka Prefecture. Patients aged ≥20 years who experienced OHCA caused by suffocation that occurred before the arrival of emergency-medical-service (EMS) personnel were included. Patient characteristics, prehospital interventions, and outcomes were compared based on the cause of suffocation (rice cake and non-rice-cake). The primary outcome was 1-month survival after OHCA.

**Results:**

In total, 46 911 adult OHCAs were observed during the study period. Of the OHCAs, 7.0% (3,294/46,911) were due to suffocation, with choking due to rice cake as the cause in 9.5% of cases (314/3,294), and of these, 24.5% (77/314) occurred during the first 3 days of the New Year. In crude analysis, 1-month survival was 17.2% (54/314) in those with suffocation caused by rice cake and 13.4% (400/2,980) in those with suffocation due to other causes. In the multivariable analysis for all-cause suffocation, younger age, arrest witnessed by bystanders, and earlier EMS response time were significantly related to better 1-month survival.

**Conclusion:**

Approximately 10% of OHCAs due to suffocation were caused by rice-cake choking, and 25% of these occurred during the first 3 days of the New Year. Further efforts for establishing preventive measures as well as improving the early recognition of choking and encouraging bystanders to call EMS sooner are needed.

## INTRODUCTION

More than 120 000 out-of-hospital cardiac arrests (OHCAs) are documented in Japan every year,^1^ and OHCA is recognized as a national public health problem. Approximately 40% of OHCAs were of non-cardiac origin, and 20% of them were caused by suffocation.^2^ Although numerous studies have been conducted to examine prognostic factors for OHCAs of cardiac origin, non-cardiac OHCAs, including those due to suffocation, have not been studied in detail.

More than 9000 deaths are reported annually in Japan due to accidental suffocation, and half of these deaths are attributed to food choking.^3^ The most common cause of food-choking accidents in Japan is rice cake, or “*mochi*,”^4^ which is a Japanese traditional food made of glutinous rice pounded into paste and molded into shape. While it is eaten throughout the year, it is most commonly sold and consumed during the New Year holidays. Since it is highly cohesive and adhesive, deaths due to suffocation caused by rice cake consumption frequently occur every year in Japan, especially among elderly people. According to a report by the Tokyo Fire Department, between 2010 and 2014, more than 100 people were sent to the hospital due to rice-cake-choking accidents each year in Tokyo, and 7.5% of them died before the first medical examination.^5^ Considering the elderly population has been rapidly increasing in recent years in Japan, it is necessary to evaluate and understand the characteristics and factors related to outcomes after OHCAs caused by rice cake-related choking in order to plan appropriate countermeasures to prevent these accidents in the community setting.

A prospective population-based registry of OHCA in Osaka Prefecture, the Utstein Osaka Project, covers a population of approximately 8.8 million people and consecutively registers all OHCA cases according to the Utstein-style guidelines.^6^^,^^7^ Using this database, the present study aimed to investigate the characteristics of patients, actual situation of prehospital interventions, and outcomes after OHCA due to suffocation, with a particular focus on suffocation caused by Japanese rice-cake choking.

## METHODS

### Study setting and EMS system in Osaka Prefecture

Osaka Prefecture is the third largest prefecture in Japan, having 8,865,245 residents in 2010 in an area of 1892 km^2^.^8^ Details of the emergency medical service (EMS) system in Osaka Prefecture have been described previously.^2^^,^^9^ Briefly, Osaka has 34 fire stations with emergency dispatch centers, which operate a 24-hour EMS system. When someone calls the number 119 (the emergency telephone number for calling the ambulance or fire department in Japan), an emergency dispatch center sends the nearest ambulance available to the site. In general, since do-not-resuscitate orders are not accepted in Japan, termination of resuscitation on site by EMS personnel is not permitted. Therefore, excluding cases of decapitation, incineration, decomposition, rigor mortis, or dependent cyanosis, all OHCA patients treated by EMS personnel are transported to hospitals.

### Data collection

The Utstein Osaka Project is a large prospective population-based cohort study of OHCA covering all of Osaka Prefecture. Data registration in this project has been conducted based on the Utstein-style guidelines, which are utilized worldwide as a standardized reporting guideline for cardiac arrest.^6^^,^^7^ Cardiac arrest is defined as the cessation of cardiac mechanical activity as confirmed by the absence of signs of circulation.^6^^,^^7^ The arrest is presumed to be of cardiac origin unless an apparent non-cardiac cause is confirmed, including external causes (suffocation, hanging, fall, drowning, traffic injury, drug overuse, and unclassified external causes), respiratory disease, malignant tumor, or stroke according to the hospital medical records.^6^^,^^7^ For external causes, when the cause of OHCA is suffocation due to rice cake-related choking, a “*mochi*” flag is to be set on the input item. These diagnoses were defined clinically by the physicians in a hospital and/or EMS personnel at the scene. All input data forms were transferred and integrated into the registration system at the Information Center for Emergency Medical Services of Osaka. The data were then checked by the investigators, and when incomplete data are found, EMS personnel in charge were asked to complete the data form. In the present study, we obtained the following data items from the registry: age, sex, location of cardiac arrest, ability to perform activities of daily living (ADLs) before cardiac arrest, witness of cardiac arrest, first documented cardiac rhythm, defibrillations by public-access automated external defibrillators (AEDs), dispatcher instruction, type of bystander-initiated cardiopulmonary resuscitation (CPR), intravascular fluid, administration of intravascular epinephrine, advanced airway management, EMS response time (time from 119 call to EMS personnel’s contact with the patient), prehospital and total return of spontaneous circulation (ROSC) after an OHCA, hospital admission, 1-month survival, and 1-month neurological outcome. The neurological outcome was assessed by the physician in charge using the cerebral performance category (CPC) scale: category 1, good performance; category 2, moderate disability; category 3, severe cerebral disability; category 4, coma/vegetative state; and category 5, death/brain death.^6^^,^^7^

### Study subjects

In the present study, OHCA data were obtained from January 1, 2005 to December 31, 2012. Patients aged ≥20 years who suffered from an OHCA due to suffocation were included. Patients who were not attempted resuscitations by EMS personnel or bystanders and whose cardiac arrest was observed by EMS personnel were excluded.

### Outcome measures

The primary outcome measure was 1-month survival after an OHCA. The secondary outcome measures were prehospital ROSC, total ROSC, hospital admission, and 1-month survival with favorable neurological outcome. Favorable neurological outcome at 1 month was defined as CPC 1 or 2.^6^^,^^7^

### Statistical analysis

We counted the number of OHCAs due to suffocation on each day and in each month of the study period. Patient characteristics, prehospital care by EMS personnel, and outcomes of patients were compared according to the cause of suffocation (ie, rice cake consumption-related and non-rice-cake consumption-related) using an unpaired *t*-test for continuous variables and a chi-square test or Fisher’s exact test for categorical variables. In addition, we estimated the gender and age class-specific incidence rate of OHCA due to suffocation in this population. A multivariate logistic regression model was used to investigate potential factors associated with 1-month survival after OHCA, and odds ratios (ORs) and their associated 95% confidence intervals (CIs) were calculated. The explanatory variables considered in this analysis included cause of suffocation (rice cake consumption-related and non-rice-cake consumption-related), age, sex (male or female), location of cardiac arrest (home, nursing home, or other), ADL before cardiac arrest (good or disability), witnessed by bystanders (yes or no), first documented rhythm (ventricular fibrillation [VF] or non-VF), defibrillations by public-access AEDs (yes or no), dispatcher instruction (yes or no), type of bystander-initiated CPR (no CPR, compression-only CPR, or conventional CPR with rescue breathing), intravascular fluid (yes or no), administration of intravascular epinephrine (yes or no), advanced airway management (yes or no), EMS response time, and year. In this multivariable analysis, both a fully adjusted model and a model in which explanatory variables were selected by a stepwise forward selection method were considered. All tests were two-tailed, and a *P*-value of <0.05 was considered statistically significant. All statistical analyses were conducted using SPSS statistical package ver. 20.0J (IBM Corp, Armonk, NY, USA).

### Ethics

The protocol of this study was approved by the Ethics Committee of Osaka University with the assent of the EMS authorities of the local governments in Osaka Prefecture. The requirement for individual informed consent for the review of patient outcomes was waived by the Personal Information Protection Law and the national research ethics guidelines of Japan.

## RESULTS

### Occurrence of OHCAs due to suffocation

**Figure 1.**
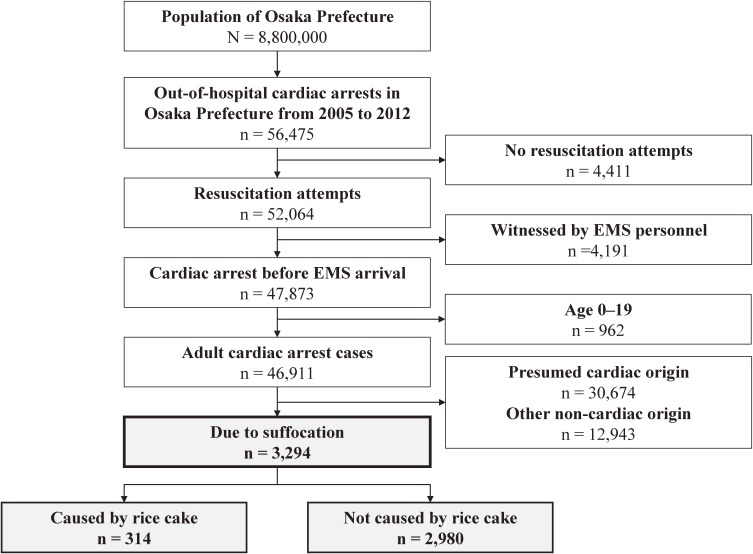
Study flow of cases of out-of-hospital cardiac arrest due to suffocation in adult patients in Osaka Prefecture between January 1, 2005, and December 31, 2012. EMS, emergency medical service.

**Figure 2.**
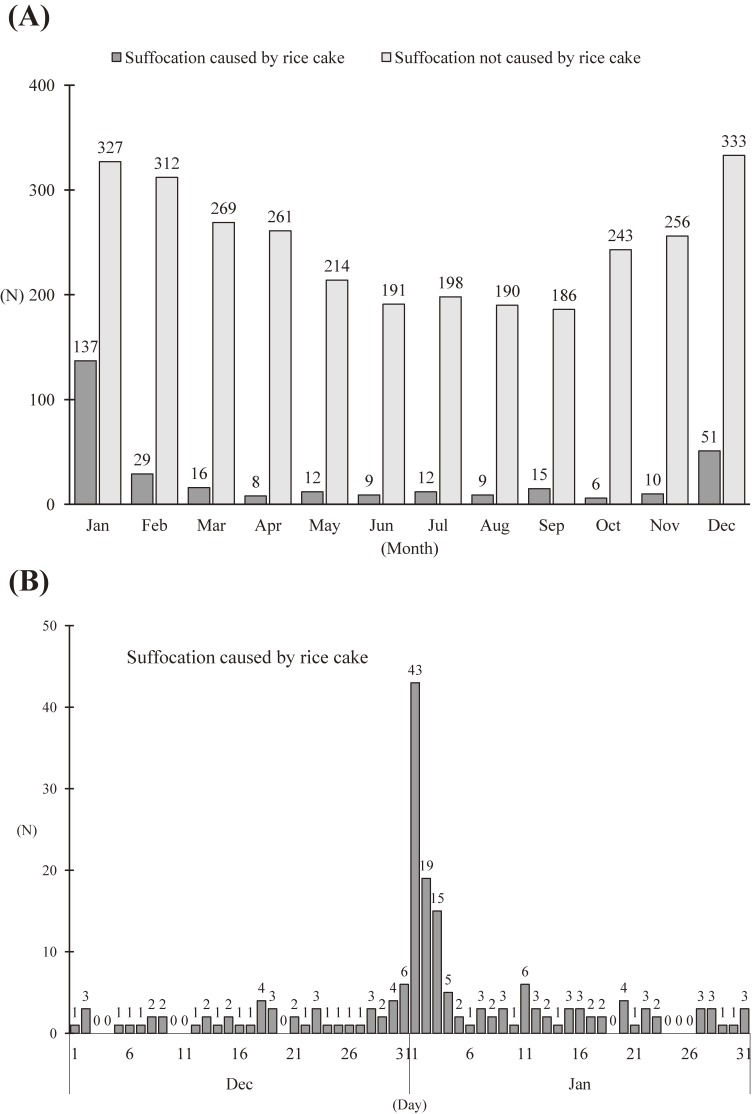
Distribution of the number of adult out-of-hospital cardiac arrest (OHCA) cases due to suffocation in Osaka Prefecture (2005–2012). (A) Monthly distribution of OHCA due to suffocation caused by rice-cake consumption and those not caused by rice-cake consumption. (B) Daily distribution of the number of OHCA cases due to suffocation caused by rice-cake choking in December and January.

Figure 1 shows the flow of OHCA due to suffocation in adult patients in Osaka Prefecture during the 8-year study period. In total, 46,911 adult OHCAs were observed during the study period, and 7.0% of these (3,294/46,911) were due to suffocation. Among these patients, the cause of suffocation was rice-cake choking in 9.5% (314/3,294) while 90.5% (2,980/3,294) were due to causes other than rice cake-related choking. Figure 2 shows the distribution of the number of OHCAs due to suffocation in adult patients during the study period. Generally, OHCAs due to suffocation were most likely to occur during the winter (Figure 2A). This tendency is particularly evident among OHCAs caused by rice-cake choking, as 16.2% (52/314) occurred in December, 43.6% (137/314) in January, and 9.2% (29/314) in February. In terms of the daily distribution, cases of OHCA caused by rice-cake choking were concentrated in the first 3 days of the New Year, as 24.5% (77/314) of all OHCAs caused by rice-cake choking occurred during those days (Figure 2B). Table 1 shows the incidence rate of OHCA due to suffocation in Osaka Prefecture during the study period. The overall incidence rate of OHCA due to suffocation was 45.79 per 100,000 population per year, and that of suffocation caused by rice cake was 4.36.

**Table 1.** Incidence rate of out-of-hospital cardiac arrest due to suffocation in Osaka Prefecture

Gender	Age, years	Incidence rate per 100 000 population per year		

		Caused by rice cake	Not caused by rice cake	Total
Male	20–39	0.00	5.03	5.03
	40–59	0.73	11.93	12.65
	60–79	11.29	59.60	70.89
	≥80	58.55	425.35	483.90
	Total	6.22	42.11	48.33

Female	20–39	0.09	2.76	2.84
	40–59	0.80	7.69	8.49
	60–79	3.83	33.34	37.17
	≥80	15.34	342.05	357.39
	Total	2.68	40.80	43.48

Total	20–39	0.04	3.88	3.92
	40–59	0.76	9.78	10.54
	60–79	7.34	45.70	53.04
	≥80	29.65	369.65	399.30
	Total	4.36	41.42	45.79

### Patient characteristics and prehospital care by EMS personnel

Table 2 shows the patient and EMS characteristics of OHCAs due to suffocation. In total, the majority of patients (80.6% of OHCAs caused by rice-cake choking and 78.5% of those not caused by rice-cake choking) were elderly people aged ≥70 years. OHCAs caused by rice-cake choking were more common in male patients (67.8%), and mostly occurred at home (87.9%). Overall, approximately 70% of OHCAs were witnessed by bystanders, but VF as the initial cardiac rhythm (1.9%) and defibrillation by public-access AED (0.3%) were rare. The proportion of bystander initiated-CPR was significantly lower among OHCAs caused by rice-cake choking (29.9% [*n* = 94] received bystander CPR) than among those due to other causes (50.4% [*n* = 1,502] received bystander CPR).

**Table 2.** Patient and EMS characteristics of out-of-hospital cardiac arrest cases due to suffocation caused by rice cake and those not caused by rice cake

	Total	Caused by rice cake	Not caused by rice cake	*P*
		
	(*n* = 3,294)	(*n* = 314)	(*n* = 2,980)	
Age, years, median (IQR)	81 (72–88)	78 (72–85)	81 (72–88)	<0.001
Age group, *n* (%)				<0.001
Aged 20–69 years	702 (21.3%)	61 (19.4%)	641 (21.5%)	
Aged 70–79 years	801 (24.3%)	120 (38.2%)	681 (22.9%)	
Aged 80–89 years	1,124 (34.1%)	97 (30.9%)	1,027 (34.5%)	
Aged ≥90 years	667 (20.2%)	36 (11.5%)	631 (21.2%)	
Men, *n* (%)	1,656 (50.3%)	213 (67.8%)	1,443 (48.4%)	<0.001
Location of arrests, *n* (%)				<0.001
Home	1,956 (59.4%)	276 (87.9%)	1,680 (56.4%)	
Nursing home	1,039 (31.5%)	18 (5.7%)	1,021 (34.3%)	
Other	299 (9.1%)	20 (6.4%)	279 (9.4%)	
Good activities of daily living before arrest, *n* (%)	1,372 (41.7%)	208 (66.2%)	1,164 (39.1%)	<0.001
Witnessed by bystanders, *n* (%)	2,230 (67.7%)	219 (69.7%)	2,011 (67.5%)	0.415
First documented rhythm, *n* (%)				0.097
Ventricular fibrillation	63 (1.9%)	4 (1.3%)	59 (2.0%)	
Pulseless electrical activity	1,161 (35.2%)	130 (41.4%)	1,031 (34.6%)	
Asystole	1,990 (60.4%)	172 (54.8%)	1,818 (61.0%)	
Unknown	80 (2.4%)	8 (2.5%)	72 (2.4%)	
Shock by a public-access AED, *n* (%)	9 (0.3%)	1 (0.3%)	8 (0.3%)	0.872
Dispatcher instruction, *n* (%)	1,664 (50.5%)	181 (57.6%)	1,483 (49.8%)	0.008
Bystander-initiated CPR, *n* (%)				<0.001
No CPR	1,698 (51.5%)	220 (70.1%)	1,478 (49.6%)	
Compression-only CPR	965 (29.3%)	66 (21.0%)	899 (30.2%)	
Conventional CPR with rescue breathing	631 (19.2%)	28 (8.9%)	603 (20.2%)	
Intravenous fluid, *n* (%)	692 (21.0%)	64 (20.4%)	628 (21.1%)	0.775
Epinephrine, *n* (%)	428 (13.0%)	37 (11.8%)	391 (13.1%)	0.503
Advanced airway management, *n* (%)	2,160 (65.6%)	231 (73.6%)	1,929 (64.7%)	0.002
EMS response time (call to contact with a patient) (min, median [IQR])	7 (6–9)	7 (6–9)	7 (6–9)	0.154
Hospital arrival time (call to hospital arrival) (min, median [IQR])	28 (23–34)	28 (23–33)	28 (23–34)	0.333
Year, *n* (%)				0.214
2005	360 (10.9%)	34 (10.8%)	326 (10.9%)	
2006	374 (11.4%)	35 (11.1%)	339 (11.4%)	
2007	369 (11.2%)	21 (6.7%)	348 (11.7%)	
2008	454 (13.8%)	43 (13.7%)	411 (13.8%)	
2009	419 (12.7%)	38 (12.1%)	381 (12.8%)	
2010	409 (12.4%)	41 (13.1%)	368 (12.3%)	
2011	441 (13.4%)	49 (15.6%)	392 (13.2%)	
2012	468 (14.2%)	53 (16.9%)	415 (13.9%)	

AED, automated external defibrillator; CPR, cardiopulmonary resuscitation; EMS, emergency medical service; IQR, interquartile range.

### Outcomes after OHCA due to suffocation

Table 3 shows the outcomes after OHCA due to suffocation. In general, better outcomes were observed among patients with OHCAs due to suffocation caused by rice cake than among those not caused by rice cake. In crude analysis, the proportions of patients with 1-month survival after an OHCA were 17.2% (54/314) among those caused by rice cake and 13.4% (400/2,980) among those not caused by rice cake. Table 4 shows the factors related to 1-month survival after OHCA due to all causes of suffocation. In the fully adjusted multivariable analysis, younger age (adjusted OR for 1-year increment, 0.98; 95% CI, 0.97–0.99), arrest witnessed by bystanders (adjusted OR 5.26; 95% CI, 3.85–7.14), intravenous fluid administration (adjusted OR 1.76; 95% CI, 1.26–2.46), and earlier EMS response time (adjusted OR for 1-minute increment, 0.88; 95% CI, 0.84–0.92) were significantly related to better survival, whereas cause of suffocation and initiation of bystander CPR did not show a significant relationship. The results were similar when we used the model with a stepwise forward selection method (Table 4).

**Table 3.** Outcomes of out-of-hospital cardiac arrest due to suffocation caused by rice cake and those not caused by rice cake

	Total	Caused by rice cake	Not caused by rice cake	*P*
		
	(*n* = 3,294)	(*n* = 314)	(*n* = 2,980)	
One-month survival, *n* (%)	454 (13.8%)	54 (17.2%)	400 (13.4%)	0.065
Pre-hospital ROSC, *n* (%)	577 (17.5%)	66 (21.0%)	511 (17.1%)	0.086
Total ROSC, *n* (%)	2,035 (61.8%)	226 (72.0%)	1,809 (60.7%)	<0.001
Hospital admission, *n* (%)	1,863 (56.6%)	210 (66.9%)	1,653 (55.5%)	<0.001
CPC 1 or 2, *n* (%)	85 (2.6%)	13 (4.1%)	72 (2.4%)	0.068

CPC, cerebral performance category; ROSC, return of spontaneous circulation.

**Table 4.** Factors related to 1-month survival after out-of-hospital cardiac arrest due to suffocation

	One-month survival		Crude OR		Adjusted OR^b^		Adjusted OR^c^	
			
	*N*	*n* (%)	OR (95% CI)	*P*	OR (95% CI)	*P*	OR (95% CI)	*P*
Cause of suffocation								
Rice cake-related	314	54 (17.2%)	1.34 (0.98–1.83)	0.066	1.26 (0.90–1.77)	0.183		
Non-rice cake-related	2,980	400 (13.4%)	Ref.		Ref.			
Age, 1-year increments		—	0.99 (0.98–0.99)	<0.001	0.98 (0.97–0.99)	<0.001	0.98 (0.97–0.98)	<0.001
Gender								
Male	1,656	238 (14.4%)	Ref.		Ref.			
Female	1,638	216 (13.2%)	0.91 (0.74–1.10)	0.324	1.13 (0.91–1.40)	0.280		
Location of arrests								
Home	1,956	273 (14.0%)	Ref.		Ref.			
Nursing home	1,039	121 (11.6%)	0.81 (0.65–1.02)	0.075	0.94 (0.71–1.24)	0.651		
Other	299	60 (20.1%)	1.55 (1.13–2.11)	0.006	1.37 (0.97–1.94)	0.072		
Good activities of daily living before arrest								
Good	1,372	198 (14.4%)	1.10 (0.90–1.33)	0.361	1.04 (0.83–1.32)	0.709		
Disability	1,922	256 (13.3%)	Ref.		Ref.			
Witnessed by bystanders								
No	1,064	51 (4.8%)	Ref.		Ref.		Ref.	
Yes	2,230	403 (18.1%)	4.35 (3.23–5.88)	<0.001	5.26 (3.85–7.14)	<0.001	5.13 (3.76–7.01)	<0.001
First documented rhythm^a^								
Non-VF	3,231	453 (14.0%)	N.A.		N.A.		N.A.	
VF	63	1 (1.6%)						
Shock by a public-access AED^a^								
No	3,285	453 (13.8%)	N.A.		N.A.		N.A.	
Yes	9	1 (11.1%)						
Dispatcher instruction								
No	1,630	245 (15.0%)	Ref.		Ref.			
Yes	1,664	209 (12.6%)	0.62 (0.50–0.78)	<0.001	1.06 (0.85–1.33)	0.577		
Bystander-initiated CPR								
No CPR	1,698	268 (15.8%)	Ref.		Ref.			
Compression-only CPR	965	108 (11.2%)	0.67 (0.53–0.85)	0.001	0.78 (0.59–1.03)	0.082		
Conventional CPR with rescue breathing	631	78 (12.4%)	0.75 (0.57–0.99)	0.040	0.95 (0.69–1.30)	0.727		
Intravenous fluid								
No	2,602	325 (12.5%)	Ref.		Ref.		Ref.	
Yes	692	129 (18.6%)	1.61 (1.28–2.01)	<0.001	1.76 (1.26–2.46)	0.001	1.56 (1.24–1.97)	<0.001
Epinephrine								
No	2,866	371 (12.9%)	Ref.		Ref.			
Yes	428	83 (19.4%)	1.62 (1.24–2.11)	<0.001	0.90 (0.61–1.34)	0.603		
Advanced airway management								
No	1,135	159 (14.0%)	Ref.		Ref.			
Yes	2,160	295 (13.7%)	0.97 (0.79–1.19)	0.774	0.81 (0.65–1.02)	0.069		
EMS response time (call to contact with a patient) (one minute increment)		—	0.88 (0.85–0.92)	<0.001	0.88 (0.84–0.92)	<0.001	0.88 (0.84–0.92)	<0.001
Year		—	1.01 (0.97–1.05)	0.674	1.02 (0.97–1.07)	0.524		

AED, automated external defibrillator; CPR, cardiopulmonary resuscitation; EMS, emergency medical service; IQR, interquartile range.

^a^These variables were not included in the logistic regression analyses as the number was too small.

^b^The explanatory variables included all variables listed above.

^c^Age, Witnessed by bystanders, Intravenous fluid, and EMS response time were selected as explanatory variables by a stepwise forward selection method.

## DISCUSSION

Using the prospective population-based registry of OHCAs in Osaka Prefecture, we described the epidemiology of OHCAs due to suffocation, with a specific focus on suffocation caused by Japanese rice-cake consumption. Approximately 10% of OHCAs due to suffocation were caused by rice-cake choking, and one-fourth of them occurred during the first 3 days of the New Year. In addition, the majority of the patients were elderly people. Although suffocation is the most common cause of non-cardiac OHCA^2^ and rice cake is a food with a well-known high-risk of choking for elderly people in Japan, the patient characteristics, prehospital interventions, and outcomes after an OHCA due to suffocation in prehospital setting have been poorly understood. Therefore, our findings should provide valuable clues for the development of countermeasures to prevent the occurrence of unexpected death due to suffocation in the upcoming super-aging era.

Our results showed that OHCAs due to suffocation mainly occurred in the winter, and those caused by rice-cake choking were strikingly concentrated in the first 3 days of the New Year. One research paper reported that the incidence of OHCA of non-cardiac origin was increased and significantly clustered around New Year’s Day in Japan but did not address the cause of this unexpected increase.^10^ Our findings indicated that one factor contributing to this increase in the incidence of OHCA was the rice-cake choking accidents that occurred on those days. Japanese rice cake is an important feature of the New Year’s holidays for Japanese people. According to the Ministry of Internal Affairs and Communications, the mean yearly volume of rice cake purchased by households was 2,437 g (799 g per person) in 2013,^11^ most of which is eaten during the first week of the New Year. Since rice cake is easy to choke on, especially for elderly people with preexisting difficulty in mastication/swallowing, Japanese authorities warn people about the risk of choking every year. For example, the Tokyo Fire Department advises people to cut the rice cakes into small pieces, chew them slowly, and learn how to perform basic first aid.^5^ Nevertheless, many victims go to the hospital every year because of suffocation due to rice cake; this has also been reported outside Japan.^12^^,^^13^

The outcomes after an OHCA among our target population were relatively better than those after OHCA of other non-cardiac causes, which were reported in a previous study.^2^ This may be because ischemic damage to the heart is less severe in cardiac arrest due to suffocation than that in OHCA of other origins, as shown in an animal study.^14^ In addition, considering that it generally takes several minutes for a cardiac arrest to develop after respiratory arrest by choking, the time-interval from cardiac arrest to EMS arrival might have been relatively shorter than for OHCAs of other origin.

On the other hand, the results of our multivariable analysis showed that bystander-initiated CPR was not associated with 1-month survival, whereas the variables of having the arrest witnessed by bystanders and an earlier EMS response time were both significantly related to better survival. As has also been shown in a previous study,^2^^,^^15^ the impact of bystander-initiated CPR on survival after non-cardiac OHCA is considered to be small, although early CPR is a key component of the chain of survival. Thus, this finding also emphasizes the importance of efforts to prevent the occurrence of suffocation in the high-risk elderly population and among those with dysphagia, including swallowing therapy, dietary modification, and oral care.^16^^,^^17^ In addition, further efforts to inform the public of the risk of cardiac arrest due to suffocation could lead to earlier recognition of choking by bystanders and to people calling 119 earlier.

A previous study suggested that prehospital Magill forceps use by EMS personnel improves outcomes of OHCA patients with airway obstruction due to a foreign body,^18^ but this procedure generally requires temporary suspension of CPR. Therefore, removal of foreign body by bystanders before EMS arrival should play an important role in improving outcomes after OHCA due to suffocation. Several studies have suggested that bystanders tend to attempt to clear the airway rather than perform standard CPR.^19^^,^^20^ Considering the low proportion of administration of bystander CPR in OHCAs caused by rice cake choking (approximately 30%) in this study, many bystanders might have tried to remove food material from the victim’s mouth without performing CPR. Therefore, proper basic life support-related education for caregivers of elderly people with dysphagia, including abdominal thrust, back blow, and chest thrust methods, is needed,^21^ so that they can expeditiously remove foreign bodies and start CPR before EMS arrival.

The multivariable analysis for all-cause suffocation also suggested that intravenous fluid administration was related to better 1-month survival. According to previous studies,^22^^,^^23^ the effect of intravenous fluid administration for OHCA patients in the prehospital setting is still under debate. One study reported that intravenous fluid administration for OHCAs, of both cardiac and non-cardiac origins, was not effective with regard to improved outcome,^22^ whereas another study reported that intravenous access was associated with a reduction in hospital mortality in non-injured, non-cardiac-origin arrest patients.^23^ Therefore, further research should confirm the effectiveness of advanced life support by EMS personnel, including intravenous fluid administration.

### Limitations

This study has some limitations. First, this study focused on OHCA due to suffocation caused by rice cake, and the etiologies of suffocation due to causes other than rice cake choking were unclear, which makes it difficult to interpret the results of cases of OHCA due to non-rice-cake-related choking. These cases may include suffocation due to inhalation of gastric contents, inhalation and ingestion of other objects causing obstruction of the respiratory tract, other specified/unspecified threats to breathing, as well as other food-choking accidents,^3^ but we did not obtain information on them. Therefore, for example, we were not able to address the reason of the observed increase in the number of OHCAs due to suffocation not caused by rice cake in the winter season. Second, we did not obtain information on several background factors that could affect the occurrence and outcomes of OHCAs (such as past medical history, medication, life habits, and socio-economic status) and did not address potential variability in post-arrest care (such as hemodynamic support, induced hypothermia, and coronary interventional therapies).^24^ For example, several studies reported that the majority of victims who died from food choking had underlying conditions that adversely affected mastication/swallowing, such as dementia.^20^^,^^25^ In addition, information on whether or not bystanders attempted to remove foreign bodies was also not available. Therefore, although our multivariable analyses did not show a significant difference in the outcome between OHCAs caused by rice-cake choking and those not caused by rice cake, the point estimate may have been further attenuated toward the null if we were able to control residual confounding factors more completely. Third, our study area was limited to one prefecture; hence, the results may not be generalizable to other areas. Considering that the marketing strategies, consumption, cooking methods of rice cake, and other environmental circumstances may vary according to regional customs, the pattern and incidence of suffocation could differ by prefecture. Therefore, further investigations using data from other prefectures are needed to confirm our findings. Fourth, data uncertainty, validity, integrity, and ascertainment bias were also possible sources of bias in this study. However, the large sample size, covering all OHCAs in Osaka Prefecture, and the adoption of the standardized data format based on the Utstein-style reporting guidelines^6^^,^^7^ would have minimized such potential bias.

### Conclusion

Approximately 3,300 OHCAs due to suffocation occurred in Osaka Prefecture during the 8-year study period, and 10% of these were caused by Japanese rice cake, or “*mochi*.” One-fourth of cases of OHCAs caused by rice-cake choking occurred during the first 3 days of the New Year. More efforts for establishing preventive measures, as well as giving proper basic life support education, improving the early recognition of choking, and encouraging bystanders to call EMS sooner, are needed.
